# Effects of Music Pitch and Tempo on the Behaviour of Kennelled Dogs

**DOI:** 10.3390/ani11010010

**Published:** 2020-12-23

**Authors:** Veronica Amaya, Kris Descovich, Mandy B. A. Paterson, Clive J. C. Phillips

**Affiliations:** 1 School of Veterinary Sciences, University of Queensland, White House Building (8134), Gatton Campus, Gatton, QLD 4343, Australia; k.descovich1@uq.edu.au (K.D.); mpaterson@rspcaqld.org.au (M.B.A.P.); 2 Royal Society for the Prevention of Cruelty to Animals, Queensland, Wacol, QLD 4076, Australia; 3 Curtin University Sustainability Policy (CUSP) Institute, Curtin University, Perth, WA 6845, Australia; clive.phillips@curtin.edu.au

**Keywords:** canine, arousal, alertness, white noise

## Abstract

**Simple Summary:**

Domestic dogs often live in confined environments for different reasons. These environments can be challenging for some dogs and this can lead to high levels of stress and arousal, which could affect welfare. Auditory enrichment has been shown to reduce arousal-related behaviours in dogs, and the aim of this study was to investigate if these effects are induced by particular characteristics of music such as tempo and pitch. The behaviour of 10 kennelled dogs was monitored in response to music tracks played with different characteristics (high pitch, low pitch, fast tempo and slow tempo), as well as white noise and a control. Low pitch tracks seemed to prompt behavioural changes by increasing the level of alertness of the dogs, potentially due to the association between low frequency vocalisations with agonistic contexts, making them more vigilant of their surroundings.

**Abstract:**

Confinement can be stressful for some dogs and this can lead to behavioural issues and poor welfare. A key component of the stress response is behavioural arousal, characterised by increased alertness and sensory sensitivity. This makes behavioural observations a useful tool to assess stress, as they provide insight into an animal’s internal state. Auditory enrichment has been shown to reduce arousal-related behaviour in dogs, but it is not clear if specific characteristics of a music track, such as tempo and/or pitch, produce these effects. The aim of this study was to compare behavioural responses of dogs to music tracks played with different characteristics (high pitch, low pitch, fast tempo, and slow tempo), as well as white noise and a control. Pitch and tempo modifications were applied to ten piano music songs and the six treatments (four different treatment-song combinations, white noise, and control) were presented daily, for ten minutes each, to ten dogs over ten days. Behavioural changes seemed to be driven by low-pitch tracks, which increased the level of alertness of the dogs. These findings could be related to the Morton’s motivations-structural rules: harsh, low frequency vocalisations signal aggressive motivations in mammals. Dogs may have perceived low-pitch tracks as more unsettling and were therefore more active and alert when listening to them.

## 1. Introduction

Domestic dogs (*Canis familiaris*) are often housed in confined conditions such as in shelters, laboratories, university facilities, and boarding kennels, amongst others. These environments can be loud and unpredictable, which is challenging for many dogs [1]. This can lead to high levels of stress and arousal, contributing to poor welfare [2]. A fundamental component of the stress response is behavioural arousal [3], which is characterised by increased alertness and sensory awareness in order to respond quickly to environmental stimuli [4]. Therefore, behavioural observations can be used to monitor stress in animals as they provide insight into a given situation from the animal’s perspective [5]. 

Sensory environmental enrichment, in which one or more senses of an animal are stimulated [6], can be useful to help reduce stress, and auditory stimulation is a widely used form of this type of enrichment. Music is commonly chosen as an auditory stimulus and has been used as a form of therapy due to its psychological and physiological effects [7]. It has also been shown to have positive effects in humans undergoing surgical procedures [8]. Human-based research demonstrated that music listening reduces postoperative pain, anxiety, and analgesia use, regardless of the type of music and time of exposure throughout the intervention [8]. Patients had positive effects even under general anaesthesia. 

Given that music exposure results in positive effects on humans, music has also been tested in other animal species. For example, auditory stimulation has been tested in animal shelter environments. Wells et al. [9] exposed dogs to human conversation, classical music, heavy metal music, pop music and a control (no extra auditory stimulation). They found that dogs exposed to classical music spent more time resting and vocalised less than when exposed to the other stimuli, and vocalised more when exposed to heavy metal music. Kogan et al. [10] also exposed dogs to classical and heavy metal music, as well as bespoke music specifically designed for dog relaxation, and a control (no music). In this study, the subjects were shelter dogs and short-term boarding dogs. The results show that while rescue dogs spent more time sleeping and silent than boarding dogs, there was no interaction effects between treatment and the group of dogs and both groups spent more time sleeping and less time vocalising when listening to classical music than when exposed to the other treatments. Additionally, dogs exposed to heavy metal music showed increased body shaking. Bowman et al. [11] played classical music (with low pitch and slow tempo) to shelter dogs and compared it to a control (no music). They found that when exposed to classical music, dogs spent less time barking and more time lying down. 

Further studies have also been conducted outside of the shelter environment. Puppies in training to become police working dogs [12] were exposed to either a standard socialisation protocol or one that also included additional exposure to auditory stimulation throughout the day (radio talk shows, commercial music and ambient noise such as sirens, gunshots, and car noises). Puppies exposed only to the standard socialisation protocol performed better in tests assessing reactions to handling and other human interaction. Therefore, the auditory stimuli did not seem to generate immediate significant improvements in testing results. It is possible that positive effects may have been evident when the dogs encountered challenging situations later in their life [12]. 

Auditory stimulation has also been tested in veterinary hospital settings, in anaesthetised dogs [13] and cats (*Felis catus*) [14]. Dogs were exposed to human voices at two different volumes, bespoke music specifically designed for dog relaxation and background noise (positive control), all of them + a dexmedetomidine (DM) injection. They were also exposed to background noise + a saline injection (negative control) [13]. The aim of that study was to determine the effect of auditory stimuli on the quality of DM-induced sedation. The results show that dogs were more sedated when exposed to lower levels of noise (positive control at 40–45 dB) compared to higher levels (human voice at 80–85 dB). There were no significant differences between the positive control and bespoke music treatments, suggesting that exposure to this type of music does not deepen DM sedative effects in dogs [13]. Anaesthetised female cats subjected to an ovariohysterectomy were exposed to music tracks of different genres (classical, pop and heavy metal) [14]. They had the lowest mean heart rate and systolic blood pressure values when exposed to classical music and the highest when exposed to heavy metal music, with intermediate values during pop music. 

Some studies have tested ‘species-appropriate’ auditory stimulation. Cats were exposed to music specifically designed for them, which included characteristics thought to produce affiliative and approach behaviours, as well as to music “that would be interesting and pleasant for human listeners” [15]. Cats showed more approach responses (to the speaker) and at shorter latencies to music composed for them than when human music was played. 

Another common type of auditory stimulation is white noise (“electronically produced noise in which all frequencies are represented with equal energy in each equal range of frequencies” [16]) and the effects of this have been studied in humans. Patients under spinal anaesthesia accompanied by an intravenous CNS depressant (midazolam), were exposed to either self-selected music, ambient noise, or white noise [17]. There were no significant differences in heart rate or mean arterial pressure between the three groups; however, postoperative anxiety scale scores were significantly lower in the music and white noise groups compared to those exposed to ambient noise. A similar study used the same type of anaesthesia accompanied by propofol sedation and the same auditory stimuli. Patients exposed to music required significantly less propofol to maintain sedation than patients exposed to ambient and white noise [18]. 

In summary, the effects of auditory stimulation have been tested in humans, dogs and other animals within different environments. In dogs, classical music appears to have positive behavioural and physiological effects; however, few studies have specifically investigated if these effects are induced by particular qualities of music. Two key characteristics that define music include tempo and pitch. Tempo is the speed at which a composition is performed, and in Western music this is generally measured in beats per minute (BPM) [19]. Pitch is the location of a sound in the tonal scale, relating to the speed of vibrations that a sound produces, with slow vibrations generating a low pitch and fast vibrations generating a high one. The rate per second of the vibration is known as the note’s frequency [20]. 

The aim of this study was to compare behavioural responses of kennelled dogs exposed to musical tracks modified for high pitch, low pitch, fast tempo, and slow tempo, as well as white noise and a control. We hypothesised that dogs will perform more behaviours associated with relaxation when listening to slow tempo music tracks compared to the other music variations. The same response is expected when comparing music listening with the control.

## 2. Materials and Methods 

### 2.1. Subjects 

The research subjects were 10 dogs; 6 females and 4 males, all desexed. Mean (± sd) dog age was 2.8 ± 1.4 years, ranging from 1.2 to 5 years. Five of the dogs were greyhounds, which had come originally from the racing industry. The remainder of the dogs were two Boxers, one Labrador, one Australian Kelpie and one Mastiff, which had come from the local council impounding facility. They were acquired by the University of Queensland (UQ) at different times, and the mean (± sd) time in care at study commencement was 8.6 ± 7.3 months, ranging from 1 to 26 months. These dogs were acquired by UQ for use in student handling practicals, but during this study they did not participate in any other activity. Dogs were included in the study based on availability.

### 2.2. Kennel Environment

This study was conducted at the Clinical Studies Centre of UQ at Gatton, Australia, in January 2019. Dogs were moved to a new kennel block two days prior to commencement of the study, to allow them to acclimatise to their new kennel and neighbouring dogs. The kennel block consisted of 10 individual kennels, with dimensions of 1.4 m × 2.9 m. Each kennel included a bed and an automatic refill water bowl. Both sides were solid walls that prevented visual contact between dogs and the front door was made of metallic bars, which permitted the dogs to see outside through two long windows located at the front of the kennel block. These windows were covered on the inside with black plastic to avoid dogs reacting to people and other animals walking along a ramp in front of them. Air conditioning was not used during the experimental treatments to avoid masking of the soundtracks. Each kennel had an adjacent outside run of equal area, accessible through a guillotine door, but this was closed while the treatments were applied. The dogs were taken to exercise yards during the morning cleaning and in the afternoon for toileting and exercise. They were fed dry food twice daily (from enrichment objects in the morning, e.g., carton boxes, and iceblocks in the afternoon) and had water ad libitum. No volunteers or staff members were present in the kennel block when music was played.

### 2.3. Study Design

Dogs were exposed to 10 different songs, as well as white noise and a control (ambient noise) over 10 days, divided into two blocks of 5 consecutive days, with a 2-day break in between to avoid habituation. The songs were chosen from a 51-track selection used in a previous study [21]. They were downloaded from Spotify (Stockholm, Sweden, www.spotify.com/) and had the following characteristics: a tempo of 70 or fewer BPM, valence (refers to the musical positiveness and determines how euphoric a track is) from 0 to 0.5, and energy (determines how intense a track is) of less than 0.2 (these last two were measured on scales of 0–1.0, with 0 being the lowest and 1.0 being the highest) [22]. The piano was the sole instrument used in the music, except in 2 tracks, which had violin accompaniment for small parts of the tracks. It has been suggested that single instruments require less neurological processing than multiple instruments [23]. These 10 songs were selected as they all had the same approximate starting pitch (~11025.000 Hz), and this made it possible to uniformly modify them. Audacity^®^ recording and editing software (v 2.3.0, Pittsburgh, PA, USA) was used to modify the songs. Pitch and tempo were modified choosing the ‘effect’ function and then ‘change tempo’ and ‘change pitch’ functions. Using this method, tempo changes could be made without changing pitch and vice versa. Both were increased and reduced by 30% to obtain four experimental treatments: high pitch (HP), low pitch (LP), fast tempo (FT), and slow tempo (ST). These percentages were chosen because dogs are more sensitive to frequencies between 500 Hz and 16 kHz [24]. Moreover, at these percentages, the modifications caused no, distortion in the tracks. Each track’s amplitude was averaged using the ‘normalize’ function of the Audacity software. A white noise (WN) track downloaded from YouTube account ‘Tha Secret’ (www.youtube.com/watch?v=NZs-WK3DYpQ) and a control (C) were also applied, to make a total of six treatments. Treatments and songs were randomly allocated, making sure that none of them would repeat within a single day. The order of presentation also changed between days. Each song and treatment combination was presented only once throughout the whole experiment, and white noise and the control were presented every day (Table 1).

Four speakers and two stereo sound sets (Logitech Speaker System Z623, Lausanne, Switzerland) were located in front of the kennels. They were spread out to cover the 14 m of the kennel block (they were located at 1.75, 5.25, 8.75, and 12.25 m), and they had a set volume throughout the study. The sound sets were connected to a Dell laptop (Inspiron 15-3567, Round Rock, TX, USA) where the tracks were played simultaneously to all dogs. Volume levels were checked in 6 different spots of each kennel while playing white noise (which has a stable volume) with a digital sound level meter (Digitech^®^, QM-1589, Stanford, CT, USA) to make sure the amplitude was as similar as possible in all the kennels. The average volume was 65 dB. Each treatment was played in a loop for 10 min, followed by a 20-min break until the next treatment started, for a total of a 2 h and 40 min trial each day. One minute before the beginning of a new treatment, the researcher would walk into the kennel to start the track on the laptop. The trial commencement time varied between 9:35 and 11:27 a.m., as it was dependent on when kennel cleaning was complete.

### 2.4. Data Collection and Analysis

Each kennel was fitted with two cameras (Signet^®^, QC-3694, Electus Distribution Pty. Ltd., New South Wales, Australia), one placed at the front of the kennel and one in the mesh on top of the kennel, and were set to record for the entire 2 h and 40 min per day. Focal animal sampling and continuous recording were used for state behaviours, and all-occurrence recording for event behaviours. Behaviour for every 10-min treatment period was coded by a single, experienced observer with Boris^®^ behaviour coding software (v 6.0.4. for Windows, Torino, Italy), using a standardised ethogram (Table 2). Ten videos were randomly chosen and double-coded to check intra-rater reliability. The average Cohen’s Kappa was 0.82.

### 2.5. Statistical Analysis

As the 10 dogs were simultaneously exposed to the treatments throughout the trial, behaviour data were averaged (mean) by day and time period to obtain 60 readings per behaviour (averaging individual dog’s data). A “class” dummy factor was created to distinguish between song treatments and their different modifications, and the ‘white noise’ and ‘control’ treatments. Data were statistically analysed using Minitab 18 software (Minitab. LLC, State College, PA, USA). General linear models were constructed using day, time period, class, song nested within class and treatment nested within class as fixed factors. As each song–treatment combination was presented only once throughout the experiment, there were insufficient data to estimate the interaction between these factors. Each behaviour was analysed independently. Residuals were inspected for conformity to normality using the Anderson–Darling test. A logit transformation was used for all the behaviours, as data were bounded between 0 and 600 s. Assumptions were met after transformation. Pairwise comparisons between individual treatments were analysed using a Tukey test, which adjusts for multiple comparisons. Significance was considered if *p* < 0.05, and trends were considered if *p* ≤ 0.10 but ≥ 0.05. Some behaviours were very rarely observed and therefore were not statistically analysed (door/wall pawing, pant, standing exit door, drink and excretion).

## 3. Results

### 3.1. Treatment Effects on Behaviour

Significant differences between treatments were evident in tail movement and position behaviours (Table 3). When exposed to low-pitch tracks, dogs spent more time with their tail in a medium/high position compared to slow tempo. Dogs wagged their tail more when exposed to low pitch tracks than when exposed to slow tempo tracks or the control. Conversely, when exposed to slow tempo tracks, they spent more time with their tail in a low position compared to low pitch. Dogs spent more time with their tail still when exposed to slow tempo tracks and the control than when exposed to low pitch tracks. There were trends for treatment effects on lie down total, stand, walk, and lie down-head down (*p* = 0.05, 0.05, 0.07 and 0.10, respectively). Inspection of the means suggested that these trends are largely influenced by the low pitch treatment, which had the lowest mean for lie down total and lie down-head down, and the highest mean for stand and walk of all the treatments. Regardless of the overall level of significance, when exposed to the low pitch treatment, dogs performed 11 behaviours out of 20 for either the lowest or the highest amount of time. There were no significant treatment effects for any of the other behaviours.

### 3.2. Song Effects on Behaviour

**Figure 1:**
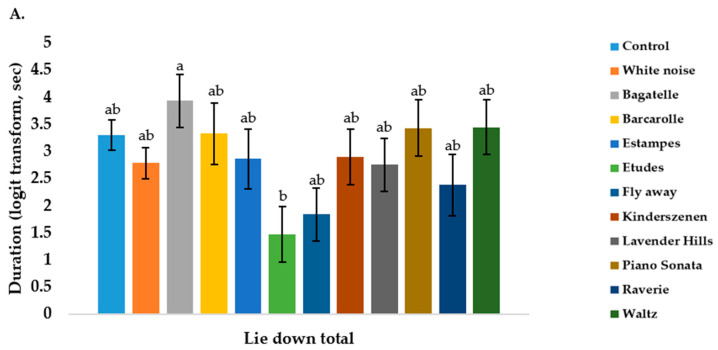

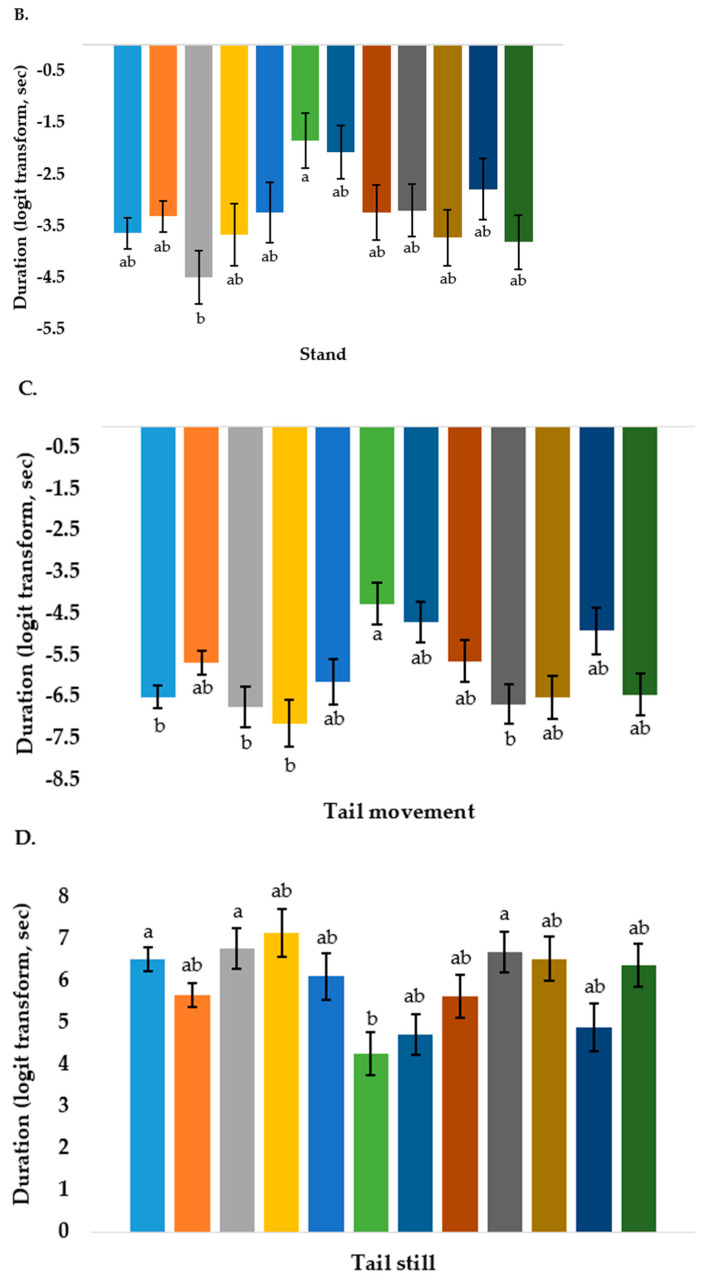
Duration of time (logit transform, seconds) dogs (*n* =10) spent (**A**) lying down, (**B**) standing, (**C**) moving their tail and (**D**)with their tail still when exposed to 10 songs, white noise and a control, for 10 min (600 s) over 10 days. Sharing different letters mean that significantly different from each other.

Some song’s effects on behaviour were evident from the data analysis. When dogs were exposed to the song Bagatelle, they lay down more and stood less than when exposed to Etudes (Figure 1A–D). Dogs wagged their tail more when listening to the song Etudes compared to Bagatelle, Barcarolle, Lavender hills, and the control, and spent less time with their tail still when listening to Etudes compared to Bagatelle, Lavender hills and the control. Non-significant behaviours are presented in Table S1.

## 4. Discussion

### 4.1. Treatment Effects on Behaviour

Behavioural changes from exposure to music seemed to be driven by low pitch tracks, increasing the level of alertness of the study dogs, as evidenced by ‘aroused’ tail movements. This finding could be related to Morton’s motivation-structural rules, that state that harsh, low-frequency vocalisations used by mammals (and birds) signal aggressive motivations [27,28]. There is a direct relationship between the frequency of a vocalisation and the size of the animal producing it: the bigger the animal, the lower the frequency of the sound that it emits, and larger animals will most likely win in a fight [27]. Contrastingly, higher frequency vocalisations signal friendly or appeasing motivations [27].

Significant differences between treatments were found in tail movement and position behaviours. When low pitch tracks were played, dogs spent more time with their tail in a medium/high position than when slow tempo tracks were played. Dogs wagged their tail more compared to slow tempo tracks and the control. Similarly, when slow tempo tracks were played, dogs spent more time with their tail in a low position compared to when low pitch tracks were played, and more time with their tail still when exposed to slow tempo tracks and the control. Tail wagging can occur in different contexts, such as play, appeasement, and aggression [25]. It is also associated with arousal [21,25,29] and with frustrating and conflict situations, for example, when an individual is fearful of other conspecifics and uses tail wagging to signal friendly intentions [30]. The dogs in this study may have used tail wagging as an appeasement signal in response to exposure to low pitch tracks. Interestingly, and contrary to what we hypothesised, dogs in the control spent more time with their tail still than when exposed to low pitch music. Considering that music has been shown to have relaxing effects in dogs, it could have been expected that dogs would wag their tail less when exposed to any of the music treatments than during the control. In a previous study with highly aroused shelter dogs, Amaya et al. [21] found that dogs in a control group (no stimuli applied) wagged their tails more than dogs exposed to piano music, and this appeared to be correlated with other behaviours associated with arousal, such as increased vocalisation, panting, and reduced time lying down with their head down. The current findings imply that the low pitch modification may have caused the different results in tail wagging between the control and the music stimuli when comparing both studies.

There was a trend for the low-pitch treatment to result in less time lying down in total and lying down with their head down, and more time standing and walking compared to all other treatments. This suggests that dogs were less relaxed and more alert when low pitch tracks were played, as resting behaviours tend to increase with reduced stress levels [10], while standing and walking indicate alertness [31]. However, these dogs were generally calm for most of the day, therefore some increase in activity and alertness would be unlikely to cause further undesirable effects, such as hyper-arousal. The increase in alertness could also be explained by the aforementioned Morton’s rules. A previous study with dogs found that barks produced in a disturbance situation (stranger ringing the bell) had a lower pitch than barks in play and isolation (from owner) situations [32]. Even humans were able to classify barks emitted in different situations, and regardless of their experience with dogs and with the specific breed (Mudi) used in the study [33]. Mudi owners, other dog owners and non-owners listened to bark playbacks from different situations (a stranger entering the garden, trainer encouraging the dog to bark aggressively and bite (‘schutzhund’), going for a walk, dog being left alone, showing the dog their favourite toy and play). They rated each bark for different kinds of emotions (aggressiveness, fearfulness, despair, playfulness, and happiness), and finally, they categorised each bark into one of the given contexts. The results show that high fundamental and peak frequencies were characteristic of barks emitted in non-aggressive situations, and low frequencies were characteristic of aggressive situations. This was in accordance with the listeners’ classification, as they gave significantly higher scores of aggressiveness to the ‘schutzhund’ and stranger situations, and significantly higher scores of despair and fear to the alone situation, showing that barks characterised by low frequencies will likely be described as aggressive. The ability of humans to recognise emotional information from barks can indicate that this type of vocalisation is also relevant for human-dog communication [33]. Dogs also seem to differentiate between conspecific vocalisations. Faragó et al. [34] exposed dogs to growl playbacks in different situations: a threatening stranger, a tug-of-war game, or food guarding while they approached a bone. Dogs withdrew from the bone mostly when food guarding growls were played. These results suggest that dogs can differentiate between the two agonistic growls, and the food guarding one produced the largest effects on behaviour because it was probably processed as contextually appropriate. There is a clear relationship between low pitch vocalisations and aggressive motivations in these studies. Even though our study did not use vocalisations, the dogs’ reactions to low pitch tracks is in accordance with the previous findings and Morton’s rules, as dogs might have deemed low pitch sounds as more aggressive signals, leading them to be more active and alert.

Slow tempo music was expected to produce the most relaxing effects in dogs; however, differences in behaviour were only found when compared to low pitch music. Previous studies have shown that different animal species seem to prefer slower tempi and it has produced positive effects in some of them. For example, chimpanzees (*Pan troglodytes*) were exposed to classical instrumental music with a tempo between 50 and 90 BPM, pop/rock music with a tempo of more than 90 BPM and silence [35]. Music was played in a target pod and results showed that the animals spent similar amounts of time in the pod during music playing and silent periods. However, the music that was playing when entering the pod had significantly slower tempo than the music that was playing when exiting it, suggesting that they preferred music with slower tempo. Similarly, McDermott et al. [36] found that tamarins and marmosets (*Callitrichidae*) had a preference for slow over fast tempi (they preferred a lullaby over techno music). However, when given the chance to choose between slow tempo lullabies and silence, they preferred the latter. Turbot fish (*Psetta maeotica*) were exposed to slow, medium, and fast tempo music, and a control (no music) [37]. The type of music used was not specified and fish were not allowed to choose their location. Fish exposed to slow tempo music had the best growth performance, while fish exposed to fast tempo music showed behavioural signs of stress (e.g., stressful swimming) and reduced food intake.

Kogan et al. [10] exposed dogs to different types of music and noted the BPM of each song to assess if any differences in behaviour were produced due to the music genre or BPM. The results show that while dogs spent more time sleeping when exposed to classical music compared to the other genre and the control (no music), no significant differences between classical music tracks were found. However, dogs spent the most time silent during the classical music track with the fastest tempo and the least during the control. Bowman et al. [11] exposed shelter dogs to classical music with low pitch and slow tempo, and found that when listening to music, they spent more time lying down and less time barking compared to when there was no music played. This differs from our findings, as we did not find significant differences in activity when dogs were exposed to slow tempo music and found a trend for low pitch music to cause dogs to be more alert. However, in the Bowman et al. [11] study, different pitches and tempi were not compared.

It could have been expected that further treatment differences in behaviour would be found, especially between slow tempo tracks, and the control. Many studies have shown that classical music exposure promotes behaviours suggestive of increased relaxation in shelter dogs (e.g., increased resting and reduced vocalisation) [9,10,11]. Bowman et al. [38] found that, when music was played, dogs spent less time standing and more time lying down (largely regardless of genre). In a previous study, Amaya et al. [21] found a reduction in arousal-related behaviours (i.e., vocalisation and panting) when shelter dogs were exposed to piano music (including the same 10 songs used in this study) compared to a control group. There are some possible explanations for why the current study results conflict with the previous literature. The dogs in this study were very placid and familiar with the kennel environment, and distractions, such as noise, around the kennels were minimal. This may have influenced the results, as dogs were already calm before exposure to treatments so changes in relaxation behaviour could have been difficult to detect. Rooney et al. [39] found that prior habituation to an environment can help in reducing stress produced by kennelling. They measured cortisol to creatinine ratios (C/C) and found that these were higher for dogs that had not been gradually habituated to a kennel compared to those that had been habituated. They concluded that novelty of the kennel environment is a major source of stress. In the present study, some of the dogs had been housed within the study environment for a considerable time upon study. Dogs had probably habituated to their environment and were more relaxed from the beginning of the trial than dogs in the shelter environment and therefore music effects on behaviour were less noticeable. Moreover, the time of stimuli presentation might have been too short to cause noticeable behaviour effects on the dogs. In the present study, dogs were exposed to the music track for 10 min each, while in a previous study in a shelter environment [21], the auditory stimulus (which included the 10 songs from the present study) was presented for 3 consecutive hours. It is also important to note that the Clinical Studies Centre is not as busy, loud, and stimulating as a shelter environment, so arousal levels are not as high as in a shelter. Another possible explanation for the difference between study findings is that the modifications made to the songs were not strong enough to cause more changes in behaviour. However, modification limits in the study were chosen because stronger changes would have caused distortion to the music tracks, which may have been uncomfortable for the dogs, and introduced other confounding variables into the study design.

White noise did not have any significant effects on kennelled dog behaviour, and this result is in line with some human studies. For example, in patients undergoing surgery under spinal anaesthesia, white noise was expected to have a masking effect over other noises and therefore help to reduce anxiety levels [17]. However, reduced anxiety was only observed during the postoperative period. Another study showed that using white noise to reduce sensory input by masking operating room noise did not decrease sedative requirements in patients undergoing surgery, while exposure to self-selected music did [18]. White noise may mask noises in some situations, but hospital and surgery environments can be very stressful, and therefore white noise might not be enough to produce relaxing effects.

### 4.2. Song Effects on Behaviour

The song Etudes was the musical piece that produced the most changes in behaviour. When compared to Bagatelle, it appeared to be more arousing for the dogs, as they spent less time lying down and more time standing and wagging their tail. When listening to Etudes, dogs also wagged their tail more than when listening to Barcarolle, Lavender hills and during the control. Etudes seemed to increase dogs’ alertness, and its tempo was slightly higher than the other songs (Table S2) but given that tempo did not produce significant effects on behaviour, no firm conclusion can be made.

## 5. Conclusions

The low pitch modification applied to the music tracks in this study appeared to produce changes in behaviour, by increasing the alertness of dogs. This could be associated to Morton’s motivation-structural rules, as also shown in previous studies, where harsh, low-pitch vocalisations are associated with aggressive motivations, and this could have made the dogs more vigilant of their surroundings. Dogs in this study were calm most of the time, therefore some increases in activity and alertness are unlikely to result in undesirable effects, such as hyper-arousal. Further studies could consider including a larger number of dogs, more breed diversity and more varied baseline behaviour, as well as increasing the treatment period times.

## Figures and Tables

**Table 1:** Song and treatment combinations played to kennelled dogs (*n* = 10) throughout the 10 days of study. Time period (600 s) corresponds to the order of presentation throughout the day.

Time Period	Day 1	Day 2	Day 3	Day 4	Day 5	Day 6	Day 7	Day 8	Day 9	Day 10
1	FT Bagatelle	C	LP Lavender hills	WN	HP Estampes	C	ST Fly away	HP Waltz	WN	LP Waltz
2	HP Raverie	WN	C	ST Kinderszenen	LP Etudes	FT Fly away	FT Kinderszenen	WN	HP Lavender hills	C
3	ST Etudes	ST Barcarolle	FT Etudes	C	FT Lavender hills	WN	HP Bagatelle	LP Fly away	C	HP Kinderszenen
4	C	LP Raverie	WN	HP Barcarolle	ST Waltz	LP Kinderszenen	LP Barcarolle	FT Barcarolle	ST Raverie	FT Raverie
5	LP Estampes	HP Piano sonata	ST Bagatelle	FT Piano sonata	WN	HP Etudes	C	ST Estampes	FT Estampes	WN
6	WN	FT Waltz	HP Fly away	LP Bagatelle	C	ST Piano sonata	WN	C	LP Piano sonata	ST Lavender hills

Treatment abbreviations: HP: high pitch, LP: low pitch, FT: fast tempo, ST: slow tempo, WN: white noise, C: control.

**Table 2:** Ethogram used for the measurement of the behaviours of dogs (*n* = 10), with descriptors and references.

Behaviour	Description	Reference (If Available)
Lie down-head up	Dog is reclining in a ventral position with its head up	
Sit	Hindquarters in contact with ground, front legs extended	[25]
Stand	Positioned with four feet in contact with ground and legs almost or fully extended	[25]
Walk	Forward movement with legs resulting in shift of whole body to a new position in enclosure	[26]
Lie down-head down	Dog is reclining in a ventral or lateral position, with a relaxed neck and head down	
Body shake	Dog shakes its whole body briefly as if drying itself	[25]
Vocalisation	Sound emitted from the mouth, often repeated in quick succession	[26]
Pant	Mouth open with tongue extended accompanied with rapid breathing	[26]
Groom	Licking behaviours directed to own body	[25]
Standing exit door	Standing on hind legs with front legs resting against the rear of the exit (at front of kennel)	[26]
Door/wall pawing	Using paws against door/wall in a digging motion	[26]
Sniff ground	Walks with nose close to ground, presumed to be sniffing it	
Object play	Any vigorous or galloping gaited behaviour directed towards a toy or other object, including chewing, biting, shaking it from side to side, batting it with a paw	[25]
Drink	Imbibe water	
Excretion	Urination or defecation	
Tail medium/high	From −30° to +90° from horizontal	
Tail low	From −30° to −90° from horizontal	
Tail movement	Tail moving in any direction and speed	
Tail still	Tail is not moving	
Front of kennel	In front third of kennel	
Middle of kennel	In middle third of kennel	
Back of kennel	In back third of kennel	
Chew/play with bedding/bed	Chew or play with bedding or bed	[25]

**Table 3:** The behaviour of dogs (*n* = 10) exposed to a control, white noise, high pitch, low pitch, fast tempo, or slow tempo treatment for 10 min (600 s) over 10 days. All of the behaviours were logit transformed. Back transformed values to seconds are reported in parentheses. When multi-variable ANOVAs were significant (*p* < 0.05), differences between individual treatments were examined using a Tukey test. Means that do not share a superscript letter are significantly different from each other.

Behaviour	C	WN	HP	LP	FT	ST	SED	F-Statistic (d.f. 3,31)	*p*-Value
Activity									
Body scratch	−6.59	−6.26	−6.57	−6.77	−6.93	−6.07	0.367	2.09	0.12
	(0.32)	(0.65)	(0.34)	(0.19)	(0.09)	(0.88)			
Body shake	−6.89	−6.80	−6.93	−6.83	−6.76	−6.79	0.068	2.19	0.11
	(0.11)	(0.17)	(0.09)	(0.15)	(0.19)	(0.17)			
Chew bedding	−6.96	−5.88	−5.93	−6.66	−6.01	−5.62	0.683	0.81	0.50
	(0.07)	(1.18)	(1.10)	(0.27)	(0.98)	(1.68)			
Groom	−5.40	−3.83	−3.86	−3.72	−4.22	−3.80	0.528	0.36	0.79
	(2.21)	(12.2)	(12.0)	(13.8)	(8.21)	(12.7)			
Lie down−head down	1.76	1.15	1.27	0.71	1.37	1.43	0.304	2.30	0.10
	(512)	(456)	(469)	(402)	(479)	(484)			
Lie down−head up	−2.16	−1.57	−1.73	−1.47	−1.88	−1.75	0.268	0.79	0.51
	(61.6)	(103)	(89.9)	(112)	(79.1)	(88.8)			
Lie down total	3.30	2.78	3.05	2.21	2.70	3.37	0.406	2.93	0.05
	(579)	(565)	(573)	(541)	(563)	(580)			
Object play	−6.03	−5.35	−5.29	−5.90	−5.16	−6.17	0.787	0.74	0.53
	(0.94)	(2.33)	(2.53)	(1.15)	(2.92)	(0.76)			
Sit	−6.43	−5.25	−5.38	−4.95	−5.70	−6.27	0.558	1.99	0.14
	(0.47)	(2.63)	(2.26)	(3.75)	(1.50)	(0.64)			
Sniff ground	−6.50	−5.71	−6.32	−6.02	−5.42	−6.29	0.352	2.87	0.05
	(0.40)	(1.48)	(0.58)	(0.96)	(2.15)	(0.61)			
Stand	−3.65	−3.32	−3.59	−2.58	−3.00	−3.68	0.424	2.93	0.05
	(14.8)	(20.6)	(15.6)	(41.7)	(28.1)	(14.3)			
Vocalisation	−5.99	−5.36	−5.97	−5.42	−6.24	−6.50	0.477	1.86	0.16
	(0.99)	(2.32)	(1.04)	(2.15)	(0.67)	(0.40)			
Walk	−4.79	−4.50	−4.67	−4.08	−4.59	−4.98	0.327	2.58	0.07
	(4.43)	(6.13)	(5.07)	(9.47)	(5.54)	(3.58)			
**Tail Position and Movement**	**C**	**WN**	**HP**	**LP**	**FT**	**ST**	**SED**	**F-Statistic (d.f. 3,31)**	***p*-Value**
**Tail low**	6.27 ^ab^	5.79 ^ab^	5.29 ^ab^	4.38 ^b^	6.15 ^ab^	6.38 ^a^	0.639	4.04	**0.02**
	(599)	(599)	(597)	(593)	(599)	(599)			
**Tail medium/high**	−6.29 ^ab^	−5.98 ^ab^	−5.30 ^ab^	−4.43 ^a^	−6.15 ^ab^	−6.39 ^b^	0.641	3.87	**0.02**
	(0.61)	(1.02)	(2.50)	(6.61)	(0.78)	(0.50)			
**Tail move**	−6.51 ^b^	−5.68 ^ab^	−5.84 ^ab^	−5.21 ^a^	−6.10 ^ab^	−6.54 ^b^	0.400	3.85	**0.02**
	(0.40)	(1.54)	(1.24)	(2.77)	(0.85)	(0.37)			
**Tail still**	6.50 ^a^	5.66 ^ab^	5.83 ^ab^	5.20 ^b^	6.07 ^ab^	6.53 ^a^	0.404	3.70	**0.02**
	(600)	(598)	(599)	(597)	(599)	(600)			
**Location in Kennel**	**C**	**WN**	**HP**	**LP**	**FT**	**ST**	**SED**	**F-Statistic (d.f. 3,31)**	***p*-Value**
Back	−3.39	−4.58	−3.67	−3.60	−4.30	−5.04	0.753	1.59	0.21
	(19.2)	(5.58)	(14.4)	(15.5)	(7.59)	(3.36)			
Front	−1.80	−2.31	−2.71	−2.03	−2.29	−2.02	0.62	0.53	0.67
	(84.9)	(53.8)	(37.0)	(69.3)	(55.0)	(70.0)			
Middle	1.28	1.83	1.60	1.41	1.56	1.53	0.426	0.07	0.98
	(469)	(517)	(500)	(483)	(496)	(493)			

Treatment abbreviations: C: control, WN: white noise, HP: high pitch, LP: low pitch, FT: fast tempo, ST: slow tempo.

## Data Availability

The data presented in this study are available on request from the corresponding author. The data are not publicly available due to University policy.

## Supplementary Materials

The following are available online at https://www.mdpi.com/2076-2615/11/1/10/s1, Table S1. The behaviour of dogs (*n* = 10) exposed to 10 songs, white noise, and a control, for 10 min (600 s) over 10 days. All of the behaviours were logit transformed. Back transformed values to seconds are reported in parentheses. When multi-variable ANOVAs were significant (*p* < 0.05), differences between individual treatments were examined using a Tukey test. Means that do not share a superscript letter are significantly different from each other; Table S2. Start and modified tempi of the 10 songs played in this study to kennelled dogs (*n* = 10) over 10 days.
