# Supplementary material for: Effects of Music Pitch and Tempo on the Behaviour of Kennelled Dogs
Source: Animals (Basel). 2020 Dec 23;11(1):10. doi: 10.3390/ani11010010 (PMC7822479; doi:10.3390/ani11010010)
Supplement: Supplementary file 1 [file animals-11-00010-s001.pdf]

animals

MDPI

Supplementary Materials:

Article

Effects of music pitch and tempo on the behaviour of kennelled dogs

Veronica Amaya, Kris Descovich, Mandy B.A. Paterson and Clive J.C. Phillips

Table S1. The behaviour of dogs (n = 10) exposed to 10 songs, white noise and a control, for 10 minutes (600 seconds) over 10 days**.** All of the behaviour were logit transformed. Back transformed values to seconds are reported in parentheses. When multi-variable ANOVAs were significant ($p < 0.05$), differences between individual treatments were examined using a Tukey test. Means that do not share a superscript letter are significantly different from each other.

Behaviour	C	WN	Piano Sonata	Bagatelle	Barcarolle	Waltz	Kinderszenen	Lavender Hills	Estampes	Raverie	Fly Away	Etudes	SED song vs C and WN	SED Song vs Song	F-statistic (d.f. 9,31)	*p*-value
Activity																
Body scratch	−6.59	−6.26	−6.09	−6.39	−6.83	−6.51	−7.00	−6.09	−6.93	−6.94	−6.39	−6.69	0.538	0.667	0.53	0.84
	(0.32)	(0.65)	(0.86)	(0.51)	(0.15)	(0.40)	(0.05)	(0.86)	(0.08)	(0.08)	(0.51)	(0.24)				
Body shake	−6.89	−6.80	−6.89	−6.70	−6.81	−6.96	−6.85	−6.65	−7.01	−6.94	−6.72	−6.77	0.099	0.122	1.85	0.10
	(0.11)	(0.17)	(0.11)	(0.24)	(0.16)	(0.07)	(0.14)	(0.28)	(0.04)	(0.08)	(0.23)	(0.19)				
Chew bedding	−6.96	−5.88	−5.85	−6.25	−6.74	−6.94	−5.33	−5.13	−6.68	−6.67	−6.90	−4.04	1.002	1.242	1.17	0.35
	(0.07)	(1.18)	(1.22)	(0.65)	(0.21)	(0.08)	(2.39)	(3.02)	(0.25)	(0.26)	(0.11)	(9.93)				
Groom	−5.40	−3.83	−2.58	−3.86	−3.79	−3.60	−4.42	−3.13	−4.25	−5.34	−4.14	−3.87	0.773	0.959	1.23	0.31
	(2.21)	(12.2)	(42.0)	(11.9)	(12.7)	(15.6)	(6.67)	(24.7)	(7.95)	(2.36)	(8.95)	(11.8)				
Lie down−head down	1.76	1.15	1.47	2.05	1.26	1.06	1.55	1.07	1.39	1.21	0.55	0.35	0.446	0.553	1.79	0.11
	(512)	(456)	(488)	(532)	(467)	(445)	(496)	(447)	(480)	(463)	(380)	(352)				
Lie down-head up	-2.16	-1.57	-1.72	-2.31	-1.57	-1.35	-2.05	-1.49	-1.81	-1.98	-1.45	-1.35	0.392	0.487	1.05	0.43
	(61.6)	(103)	(90.8)	(53.9)	(103)	(123)	(68.0)	(110)	(83.9)	(72.2)	(114)	(124)				
Object play	−6.03	−5.35	−4.61	−7.00	−5.60	−5.67	−5.83	−5.11	−6.20	−4.82	−5.65	−5.79	1.153	1.429	0.49	0.87
	(0.94)	(2.33)	(5.42)	(0.05)	(1.71)	(1.56)	(1.27)	(3.11)	(0.72)	(4.31)	(1.62)	(1.33)				
Sit	−6.43	−5.25	−6.21	−5.73	−6.18	−5.48	−6.79	−5.07	−5.55	−5.20	−4.97	−4.54	0.818	1.014	0.94	0.51
	(0.47)	(2.63)	(0.70)	(1.44)	(0.74)	(1.99)	(0.18)	(3.24)	(1.82)	(2.81)	(3.65)	(5.82)				
Sniff ground	−6.50	−5.71	−5.50	−5.87	−5.97	−6.11	−6.79	−5.80	−6.66	−6.07	−5.32	−6.04	0.515	0.639	1.17	0.35
	(0.40)	(1.48)	(1.94)	(1.20)	(1.03)	(0.83)	(0.18)	(1.32)	(0.27)	(0.89)	(2.42)	(0.93)				
Vocalisation	−5.99	−5.36	−6.53	−5.97	−6.97	−7.02	−5.84	−6.36	−6.67	−5.12	−4.70	−5.13	0.699	0.867	1.93	0.08
	(0.99)	(2.32)	(0.38)	(1.03)	(0.06)	(0.04)	(1.24)	(0.54)	(0.26)	(3.07)	(4.90)	(3.02)				
Walk	−4.79	−4.50	−4.97	−5.23	−4.92	−4.89	−4.71	−4.44	−4.84	−4.10	−4.24	−3.48	0.480	0.595	1.66	0.14
	4.43	(6.13)	(3.63)	(2.70)	(3.85)	(4.00)	(4.88)	(6.49)	(4.21)	(9.32)	(8.04)	(17.4)				

Tail Position and Movement	C	WN	Piano Sonata	Bagatelle	Barcarolle	Waltz	Kinderszenen	Lavender Hills	Estampes	Raverie	Fly Away	Etudes	SED Song vs C and WN	SED Song vs Song	F-statistic (d.f. 9,31)	*p*-value

animals

MDPI

Tail low	6.27	5.79	5.57	5.83	6.28	6.33	5.95	6.59	5.84	4.51	4.77	3.84	0.937	1.162	1.37	0.24
	(599)	(599)	(598)	(599)	(599)	(599)	(599)	(600)	(599)	(594)	(595)	(588)				
Tail medium/high	−6.29	−5.98	−5.68	−5.92	−6.22	−6.32	−5.92	−6.59	−5.93	−4.39	−4.81	−3.88	0.939	1.165	1.39	0.24
	(0.61)	(1.02)	(1.55)	(1.11)	(0.70)	(0.58)	(1.10)	(0.32)	(1.09)	(6.90)	(4.34)	(11.6)				

Location in kennel	**C**	**WN**	**Piano sonata**	**Bagatelle**	**Barcarolle**	**Waltz**	**Kinderszenen**	**Lavender hills**	**Estampes**	**Raverie**	**Fly away**	**Etudes**	**SED song vs C and WN**	**SED song vs song**	**F-statistic (d.f. 9,31)**	***p*-value**
Back	−3.39	−4.58	−5.63	−3.66	−3.96	−3.83	−4.64	−2.80	−4.76	−4.74	−3.30	−4.21	1.104	1.369	0.72	0.69
	(19.2)	(5.58)	(1.64)	(14.5)	(10.7)	(12.3)	(5.25)	(34.0)	(4.60)	(4.71)	(20.9)	(8.34)				
Front	−1.80	−2.31	−2.82	−3.46	−4.15	−1.79	−1.52	−2.52	−1.98	−1.27	−2.07	−1.02	0.911	1.130	1.70	0.13
	(84.9)	(53.8)	(33.2)	(17.8)	(8.80)	(85.2)	(108)	(44.3)	(72.2)	(131)	(66.7)	(159)				
Middle	1.28	1.83	2.12	1.50	3.47	1.09	1.81	1.46	1.02	1.40	0.59	0.79	0.624			
	(469)	(517)	(536)	(491)	(582)	(449)	(516)	(487)	(441)	(482)	(386)	(412)				

*Abbreviations: C: control, WN: white noise.

animals MDPI

Table S2. Start and modified tempi of the 10 songs played in this study to kennelled dogs (*n* = 10) over 10 days.

Artist	Song	Start Tempo (BPM)	Tempo 30% + (BPM)	Tempo 30% − (BPM)
Ludwig van Beethoven	Bagatelle, No. 25 in A Minor	67.0	87.1	46.9
Frederic Chopin	Barcarolle, in F-Sharp Major, Op. 60	57.1	74.2	39.9
Claude Debussy	Estampes, No. 2	64.3	83.5	45.0
Frederic Chopin	Etudes , Op. 10: No. 3 in E Major	68.2	88.6	47.7
Calm Children Collection	Fly away	65.7	85.4	45.9
Calm Children Collection	Lavender Hills	61.5	79.9	43.0
Ludwig van Beethoven	Piano Sonata, No. 14 in C-Sharp Minor, Op. 27 No. 2	65.8	85.5	46.0
Claude Debussy	Raverie , L. 68	66.8	86.8	46.7
Robert Schumann	Kinderszenen, Op. 15: No. 7	69.1	89.8	48.3
Frederic Chopin	Waltz, No. 3, in A Minor, Op. 34 No. 2	66.8	86.8	46.7

*Abbreviations: BPM: beats per minute.
